# Genome-wide systematic characterization of bZIP transcription factors and their expression profiles during seed development and in response to salt stress in peanut

**DOI:** 10.1186/s12864-019-5434-6

**Published:** 2019-01-16

**Authors:** Zhihui Wang, Liying Yan, Liyun Wan, Dongxin Huai, Yanping Kang, Lei Shi, Huifang Jiang, Yong Lei, Boshou Liao

**Affiliations:** 1 Key Laboratory of Biology and Genetic Improvement of Oil Crops, Ministry of Agriculture, Oil Crops Research Institute of the Chinese Academy of Agricultural Sciences, Wuhan, 430062 China; 2 Industrial Crops Research Institute, Henan Academy of Agricultural Sciences Henan Province, Zhengzhou, 450002 China

**Keywords:** bZIP gene family, Peanut, Evolution, Expression analysis

## Abstract

**Background:**

Plant basic leucine zipper (bZIP) transcription factors play crucial roles in plant growth, development, and abiotic stress responses. However, systematic investigation and analyses of the *bZIP* gene family in peanut are lacking in spite of the availability of the peanut genome sequence.

**Results:**

In this study, we identified 50 and 45 *bZIP* genes from *Arachis duranensis* and *A. ipaensis* genomes, respectively. Phylogenetic analysis showed that *Arachis bZIP* genes were classified into nine groups, and these clusters were supported by several group-specific features, including exon/intron structure, intron phases, MEME motifs, and predicted binding site structure. We also identified possible variations in DNA-binding-site specificity and dimerization properties among different *Arachis bZIPs* by inspecting the amino acid residues at some key sites. Our analysis of the evolutionary history analysis indicated that segmental duplication, rather than tandem duplication, contributed greatly to the expansion of this gene family, and that most *Arachis bZIPs* underwent strong purifying selection. Through RNA-seq and quantitative real-time PCR (qRT-PCR) analyses, the co-expressed, differentially expressed and several well-studied homologous *bZIPs* were identified during seed development stages in peanut. We also used qRT-PCR to explore changes in bZIP gene expression in response to salt-treatment, and many candidate *bZIPs* in groups A, B, and S were proven to be associated with the salt-stress response.

**Conclusions:**

This study have conducted a genome-wide identification, characterization and expression analysis of *bZIP* genes in *Arachis* genomes. Our results provide insights into the evolutionary history of the *bZIP* gene family in peanut and the funcntion of *Arachis* bZIP genes during seed development and in response to salt stress.

**Electronic supplementary material:**

The online version of this article (10.1186/s12864-019-5434-6) contains supplementary material, which is available to authorized users.

## Background

In plants, transcription factors (TFs) possess specific domains that bind upstream of target genes to regulate gene expression [1, 2]. Of these plant TFs, the basic leucine zipper (bZIP) transcription factor family is one of the largest, and was named and characterized based on the conserved bZIP domain [3, 4]. The domain is 60–80 amino acids in length and is composed of two parts: a basic region and a leucine zipper motif. The basic region is highly conserved and includes 16 amino acid residues with an invariant motif N-× 7-R/K-× 9, independently determining nuclear localization and DNA binding specificity [5, 6]. The leucine zipper motif is less conserved, and contains heptad repeats of leucine (Leu) or other bulky hydrophobic amino acids which is responsible for specific recognition and homo- and/or heterodimerization [4, 7]. The *bZIP* gene family has been systematically investigated and characterized based on the whole genome sequences of several plants, including *Arabidopsis* [4], rice [8], sorghum [9], maize [7], grapevine [10], *Brachypodium distachyon* [11], tomato [12], apple [13], cassava [14] and banana [15].

*bZIP* genes play important roles in many essential biological processes, including organ differentiation, flower and vascular development, embryogenesis, seed maturation and storage protein gene regulation [16–20]. Considerable evidence also indicates that *bZIP* genes are important regulators of signaling and the response to abiotic/biotic stress [4, 7]. The phytohormone abscisic acid (ABA) is associated with seed development as well as abiotic stress responses [21]. The ABA-responsive element binding proteins (AREB) or ABRE binding factors (ABFs), which are group A bZIP proteins, have an important role in ABA and stress signaling [22, 23]. For instance, ABI5 is involved in ABA or stress signaling to regulate seed size and development, seed germination and early seedling growth as well as response to abiotic stress [24–27]. Group B bZIP proteins, which have a transmembrane domain and a specific domain at the C-terminus, also are important to the salt stress response via endoplasmic reticulum stress signaling [28]. For example, *slbZIP38*, a group G *bZIP* gene identified in tomato, have proven to be a negative regulator of salt stress tolerance [29]. For Group S bZIP proteins, *AtbZIP1*, *MtbZIP2*, and *MtbZIP26* from *Arabidopsis thaliana* and *Medicago truncatula,* were transcriptionally induced by salt treatment, leading to an increase in salt stress tolerance [30–32]. In addition, *bZIPs* from groups C and S could cooperate with several TFs to form heterodimers and be responsible for the salt stress and seed development crosstalk network [33]. Together, these evidences indicate that *bZIP* genes have an essential role in both seed development and salt stress.

The peanut (*Arachis hypogaea*) is an important economical oilseed crop primarily grown in the tropics and semi-arid tropics and provide an important global source of vegetable oil and protein (http://faostat.fao.org/). Despite the economic and nutritional importance of peanuts, and the critical role of bZIP transcription factors in plant development and stress responses, only one *AhbZIP* gene has been reported that the over-expression of this gene (*AREB1*) is related to increase abiotic tolerance [34]. In 2016, the genomes of the two diploid ancestors (*A. duranensis* and *A. ipaensis*) of cultivated peanut have become available [35], allowing the genome-wide identification and systematic analysis of the *bZIP* gene family in *Arachis* genomes. In this study, we identified *bZIP* genes and analyzed their bZIP domain sequences, gene structure and additional MEME motifs, the DNA-binding-site specificity and dimerization properties of the bZIP proteins. We also investigated the impact of segmental and tandem duplication on the expansion of *Arachis bZIP* gene family. Using the RNA-seq and quantitative real-time PCR (qRT-PCR) methods, we analyzed their expression profiles in seed developmental stages and salt stress, and identified several candidate *Arachis bZIPs* responsive to seed development and salt stress.

## Methods

### Identification of *bZIP* genes in *A. duranensis* and *A. ipaensis* genomes

The genomic sequences of *A. duranensis* and *A. ipaensis* and their annotated gene models were downloaded from peanutbase (http://www.peanutbase.org/). BLAST were firstly conducted to search homologous *bZIP* genes using known bZIP proteins from *Arabidopsis* [4], rice [8] and maize [7] as queries. The targeting genes with similarity of E-value less than 1e-5 were retained for the following analysis. Subsequently, Hidden Markov Model (HMM) search (http://hmmer.org/) of the bZIP domain profiles (PF00170, PF07716 and PF03131) were performed to identify bZIP domain in these candidate proteins. Finally, Interpro (http://prosite.expasy.org/) and ExPASy Proteomics Server (http://prosite.expasy.org/) were used to confirm the integrity of bZIP domain in candidate genes. Each *bZIP* gene was given a unique name based on the exact position on chromosome/scaffold (from top to bottom) (Additional file 1).

### Sequence alignment and phylogenetic analysis

ClustalX 2.0 [36] were used to align the *bZIP* sequences of coding DNA and proteins from *A. thaliana*, *A. duranensis* and *A. ipaensis*. The penalties for a gap open and gap extension were 10 and 0.1, respectively. PhyML 3.0 software [37] was used for the reconstruction of the maximum likelihood (ML) phylogenetic tree. The JTT + G model were determined to be the best model for phylogenetic tree construction according to the akaike information criterion implemented in ProtTest 3.0 [38]. 100 replicates were used to produce bootstrap values. MEGA7 [39] was used to edit and show the phylogenetic tree.

### Gene structure of *bZIP* genes

The exon/intron structure of *bZIP* genes was analyzed and displayed using the GSDS platform (http://gsds.cbi.pku.edu.cn/) [40]. Genewise [41] was used to determine the correspondence on coordinates between DNA (containing exon and intron together) and protein sequences. Then, the coordinates of bZIP domain in protein sequence were transformed to that in gene sequence using in-house perl scripts. The intron splicing phase within the basic and hinge regions of bZIP domains from all *bZIP* genes were characterized and divided into different types.

### Detection of additional conserved motifs of *bZIP* genes

The MEME tool (http://meme.nbcr.net/meme/) [42] was employed to detect the additional motifs outside the bZIP domain of protein sequences. The motifs with 10–50 amino acids in length and E-value less than 1e - 40 were characterized. All the motifs were compared among *bZIP* genes to identify the group-conserved or group-specific signatures. These motifs were numbered according to their order in the protein sequences.

### Detecting duplicated genes and estimation of nonsynonymous (Ka) and synonymous (Ks) substitutions per site and their ratios

MCScan (http://chibba.agtec.uga.edu/duplication/mcscan) was used to detect the duplicated genomic segments in two *Arachis* genomes. Tandem duplication cluster was defined to contain at least two consecutive genes with sequence similarity (threshold of e < 10^− 20^), and one unrelated gene among cluster members was tolerated. The amino acid sequences of duplicated gene pairs were firstly aligned and guide the alignment of cDNA sequences in-house perl-scripts. KaKs_Calculator was used to compute Ka and Ks values of each duplicated gene pair using the YN model [43].

### Expression analysis of *Arachis bZIP* genes during seed development and under salt stress

For investigating the expression of *bZIP* genes during peanut seed development, we downloaded the previously reported RNA-seq data of peanut seeds at 20, 40 and 60 days after flowering (DAF) [44]. Trimmomatic [45] was used to check, filter or trim RNA-seq reads with low-quality. RNA-seq reads were mapped to reference genome using Hisat2 [46], and the gene expression value were estimated using RSEM [47]. DESeq2 package [48] was used for differential expression (DE) analysis.

For qRT-PCR experiment, the elite peanut cultivar ‘Zhonghua16’ was planted to collect seeds at DAF20, DAF40, and DAF60 according to the previous method [44]. For preparing salt-stress plants, 2-week-old peanut seedlings (at the four-leaf stage) were removed from the soil and hydroponically grown in a 300 mM NaCl solution (Treatment) or deionized water (Control). The time points for salt treatment were setted to be 0, 1, 5, and 10 h, and the seedling roots were collected and frozen immediately in liquid nitrogen for RNA extraction.

Total RNA was extracted with RNAprep Pure Plant Kit (TIANGEN, China) and reverse transcribed into cDNA with cDNA Synthesis Kit (Thermo Fisher Scientific, USA) following the manufacturer’s instructions. qRT-PCR were performed in a 20 μL reaction volume using a CFX connect Real-Time System (Bio-Rad, Hercules, CA, USA) and Hieff qRCR SYBR Green Master Mix (YEASEN, Shanghai, China). The peanut *Actin* gene (*Aradu.W2Y55*) was used as the internal control, and the difference in relative target gene expression among the different experimental conditions was calculated using the 2^-∆∆Ct^ method. Standard error was calculated among the three biological replicates of each experiment. Student’s *t* test was used to test the statistical significance of differences in relative target gene expression.

## Results and discussion

### Identification, phylogenetic analysis and group classification of *bZIP* genes in *A. duranensis* and *A. ipaensis*

Based on homology searches and domain verification, a total number of 50 and 45 unique *bZIP* genes were identified in *A. duranensis* and *A. ipaensis* genomes, respectively. The details for these genes, including gene ID, genomic position, domain composition, and group classification are given in Additional file 1. According to the existing nomenclature system, we assigned unique names to each of these novel *bZIP* genes: *AdbZIP1–50* and *AibZIP1–45*. After checking bZIP domains, 93 genes had a typical bZIP domain, including an invariant N-× 7-R/K motif in the basic region and a heptad repeat of Leu positioned exactly nine amino acids upstream of R/K toward the C terminus (Additional file 2). The remaining two *bZIP* genes, *AdbZIP28* and *AibZI*P22, had an unusual substitution in the basic region: a replacement of the conserved Arg/Lys (R/K) with IIe (I). This replacement has also been reported in other species [8, 49].

**Fig. 1:**
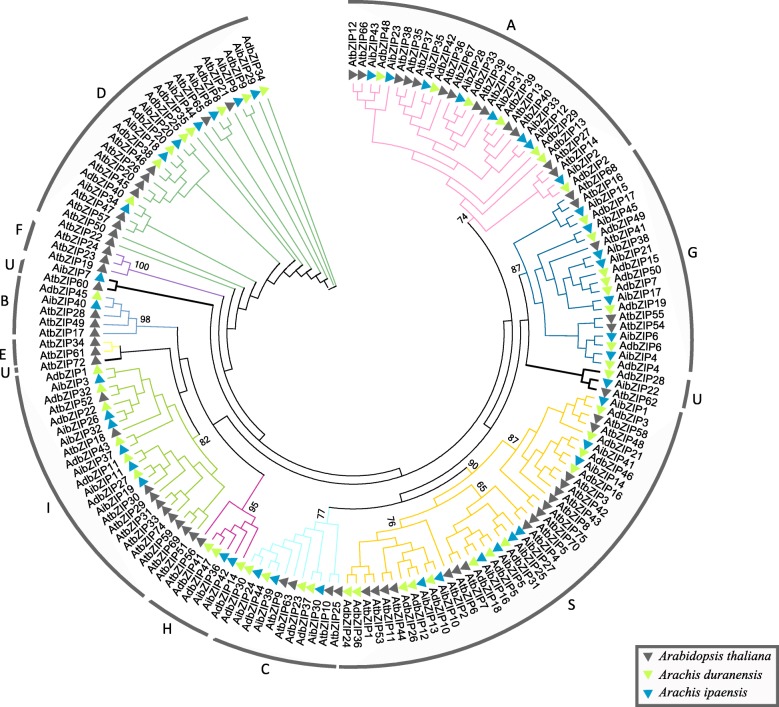
Phylogenetic analysis of peanut and Arabidopsis *bZIP* genes. Genes at branch ends from different species are denoted by different colored triangles. The peanut bZIP proteins are grouped into nine distinct clades (A–D, G–I, S, and U). bZIP protein sequences were aligned with ClustalX, and the phylogenetic tree was constructed in PhyML using the maximum likelihood method. Bootstrap values are based on 100 replicates

A systematic investigation of the *bZIP* gene family was first carried out in *Arabidopsis* [4]. In this analysis, different groups of *bZIP* genes were distinguished and named based on their phylogenetic relationships and functional divergences. This classification system has since been adopted for other species based on the clustering of *bZIP* genes from their own and *Arabidopsis* genomes [7–15, 50–53]. Here, based on a maximum likelihood (ML) analysis of bZIP proteins from *Arachis* and *Arabidopsis* genomes, we identified 11 distinct *bZIP* gene clades (groups A–I, S, and U), all with high bootstrap support (Fig. 1). The subgroup classification of *Arachis* bZIPs was further confirmed by phylogenetic tree reconstruction after adding bZIPs from soybean (Additional file 3). Most bZIP clades include closely related *Arachis* bZIPs and their *Arabidopsis* orthologs; clades E and F have no corresponding members in *A. duranensis* or *A. ipaensis*. Notably, *bZIP* genes within the same clade shared similar group-specific sequence characteristics, including exon/intron structure, intron phases, MEME motifs, and prediction of binding site structure (further analyzed below). This pattern of interspecific group clustering suggested that the group-specific features emerged prior to the divergence of *Arachis* and *Arabidopsis*. However, several differences have also accumulated in the *bZIP* genes of the different plant species over evolutionary time.

### Gene structure of *Arachis bZIP* genes

As intron and exon organization might indicate the evolutionary trajectory of *bZIP* genes [8], we examined the structure of *Arachis bZIP* genes, including intron number, length, and splicing phase (Additional file 4). We found that overall gene structures were identical or similar for *Arachis bZIPs* within the same phylogenetic group. Considering the number of introns of peanut *bZIPs,* 24% of *AdbZIP*s and 22% of *AibZIPs* were intronless, occurring exclusively in groups S and B. Among the intron-containing genes, the number of introns varied from 1 to 13 in *AdbZIP* and *AibZIP* genes. *bZIP* genes in group G had the most introns, consistent with observations in other legume genomes [32].

**Fig. 2:**
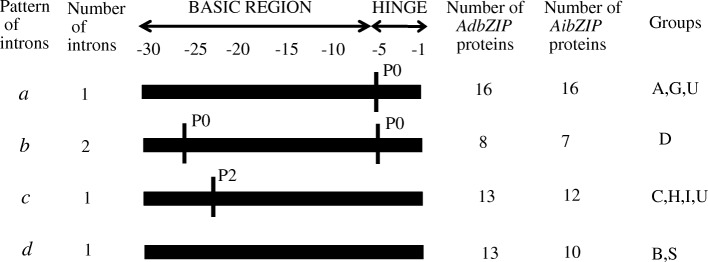
Intron patterns within the basic and hinge regions of the *Arachis* bZIP domain. The primary structure of the bZIP domain is shown at the top of the image. P0 indicates that the intron splicing site is between codons, and P2 indicates that the intron splicing site is located between the second and third nucleotides of the codon. Based on the intron incidence, intron position, and splicing phase, the *Arachis bZIP* genes exhibited four different types of patterns (**a–d**). Details of the intron positions within the bZIP domain of the peanut bZIP proteins are shown in Additional file 2

The splicing phases were designated as three splicing phases: phase 0 (P0), splicing occurred after the third nucleotide of the codon; phase 1 (P1), splicing occurred after the first nucleotide of the codon; and phase 2 (P2), splicing occurred after the second nucleotide. The phases of splicing sites within the open reading frames (ORFs) were diverse, but were highly conserved in the basic and hinge regions of bZIP domain, because any changes in these regions would affect their code and function. Based on intron position and presence or number of splicing phases in the bZIP domain, four intron patterns (*a* to *d*) in *Arachis bZIP* genes were identified (Fig. 2 and Additional file 2). Pattern *a* had just one intron inserted at the − 5 position of the hinge region, between the amino acids Gln and Ala; this pattern was identified in all *Arachis bZIP* genes in groups A and G. Pattern *b* had two intron insertions with phase 0, one in the basic region and the other in the hinge region; this pattern was identified in all *bZIP* genes in group D. Pattern *c* had a single intron inserted at the − 20 position in the basic region in phase 2 (P2), and contains all *bZIP* genes in groups C and H. Pattern *d* lacked introns in the basic and hinge regions, and includes all *bZIP* genes in groups B and S. In addition, most *Arachis bZIPs* exhibiting pattern *d* were intronless, except for *AdbZIP45* and *AibZIP40*. Each of these genes had one intron outside the basic and hinge regions. The patterns of splicing phase in *Arachis* bZIP domain observed here were consistent with those observed in other species [7, 8, 32]. The high conservation of gene structure and intron phases within phylogenetic clades supported the accepted group classification, and suggested that these different patterns of exon splicing may play an important role in functional evolution.

### The motif compositions for different groups of *Arachis* bZIPs

**Fig. 3:**
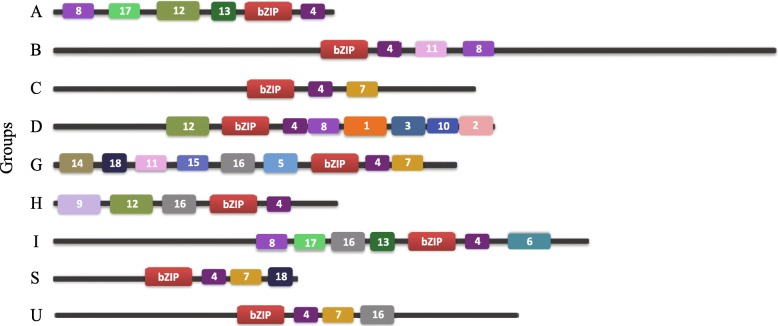
Distribution of additional conserved motifs, as identified by MEME. Motif compositions for each group of peanut bZIP proteins are shown, based on the position of the bZIP domain and additional conserved motifs outside the bZIP domain. The bZIP domains are shown in red, while other motifs are highlighted with colored boxes numbered 1 to 18. Details of the predicted conserved motifs are given in Additional file 6

In addition to the bZIP domain, many additional conserved motifs were detected in *bZIP* genes by the MEME analysis tool. As shown in Fig. 3, a total of 18 conserved motifs outside the bZIP domain were identified, and the consensus motif compositions for each subgroup were constructed (Additional file 5). These consensus motifs indicated that the overall compositions of the motifs were similar within the same subgroup but different among different groups. This suggested that functional divergence of bZIP genes may be determined by group-specific motifs. Individual examination of these motifs indicated that many were group-specific. For example, motifs 1, 2, 3, and 10 were only identified in group D; motifs 5, 14, and 15 were only identified in group G; motif 6 was only identified in group I; and motif 9 was only identified in in group H. Several motifs may be associated with specific biological functions. For example, Motif 1 is the DELAY OF GERMINATION (DOG) 1 domain, which is required for the induction of dormancy and multiple aspects of seed maturation, in part by interfering with ABA signaling components [54]. Motif 3 contains potential casein kinase II (CK II) phosphorylation sites (S/TxxD/E), which play a key role in cell division and expansion and affect diverse developmental and stress responsive pathways [55, 56]. Interestingly, these group-specific motifs have also been identified in bZIPs from the same group in other legume genomes [32], suggesting that motif composition is conserved across legume plants.

### *Arachis bZIP* DNA-binding-site structure and dimerization properties

The core basic region and the hinge region of the bZIP domain independently determine DNA-binding specificity, as demonstrated by several experiments [5, 6]. The unusual replacement of the two invariant sites, asparagine (Asn/N; position: − 18) and arginine (Arg/R; position: − 10), altered DNA-binding specificities [5]. We aligned the amino acids sequences of the basic and hinge regions of peanut bZIP proteins to identify conserved and polymorphic amino acid residues within each group (Additional file 6). No replacements of Asn/N at the − 18 position were observed in any peanut bZIPs. However, all members of group I had lysine (Lys/K) instead of arginine (R) at the − 10 position, consistent with the group I bZIPs from other legume species [32]. In addition, *AdbZIP28* and *AibZIP22* (group U) had a hydrophobic isoleucine (Ile/I) residue instead of an arginine (Arg/R), and such a replacement was demonstrated to completely inhibit the affinity of *bZIP* for AP1 in yeast [5] and does not recognize G-boxes in rice [49].

**Fig. 4:**
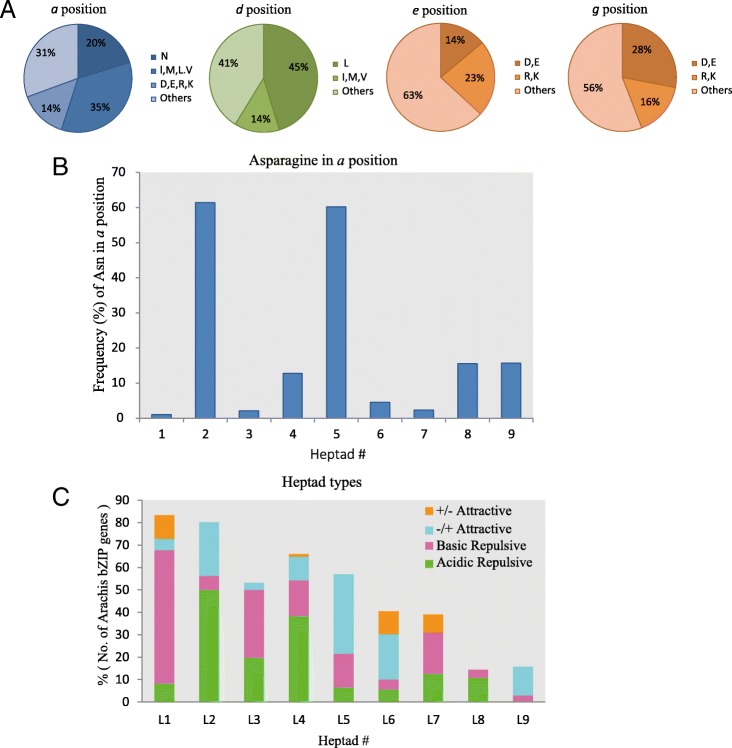
Prediction of dimerization properties of the *Arachis* bZIP proteins. **a** Pie charts indicating the frequency of various amino acids in each of the four positions (*a*, *d*, *e*, and *g*) in the Leu zipper of the *Arachis* bZIP domains. **b** Histogram of the frequency of Asn (N) in the *a* position of the Leu zipper across all *Arachis* bZIP proteins. **c** Histogram showing the frequency of attractive or repulsive *g*↔*e’* pairs per heptad across all *Arachis* bZIP proteins. The *g*↔*e*′ pairs are classified into four groups according to the electrostatic charges at the *g* and *e* positions. The +/− attractive, showed by orange box, indicates that the *g* position is basic and the following *e* position is acidic. The −/+ attractive, showed by skyblue box, indicates that the *g* position is acidic and the following *e* position is basic. The basic repulsive (pink box) and acidic repulsive (green box) indicate that the *g* and the following *e* positions have a similar charge, either both basic or both acidic

The Leu zipper sequence mediates the homo- and/or heterodimerization of bZIP proteins, which are known to bind to DNA as dimers [57, 58]. The Leu zipper region consists of heptad repeats, the amino acids are referred to *a*, *b*, *c*, *d*, *e*, *f*, and *g* within each heptad [59]. As the amino acids at the *a*, *d*, *e* and *g* positions are near the Leu zipper interface, these amino acids are the ones that primarily determine Leu zipper oligomerization, dimerization stability, and dimer specificity. We analyzed the compositions of the amino acids found at the *a*, *d*, *e* and *g* positions of peanut bZIPs (Fig. 4a).

At the *a* position, about 20% of the residues were asparagine (Asn/N), which can form a polar pocket in the hydrophobic interface, allowing for more stable N-N interactions at *a*↔*a*′ (the corresponding position in the opposite helix), as compared to other amino acids [60]. Across the different heptads, the second and the fifth heptads had the highest frequency of Asn/N residues in the *a* position (61.46 and 60.22%, respectively; Fig. 4b). At the *d* position (Fig. 4a), the Leu was found in 45% of all peanut bZIPs and is one of the most dimer-stabilizing aliphatic amino acids [61]. At the *e* position, 37% of all peanut bZIPs had acidic amino acids D or E, while at the *g* position, 44% of all peanut bZIPs had the basic amino acids R or K (Fig. 4a). These charged amino acids are thought to form salt bridges between helices in electrostatic interactions [62]. The attractive or repulsive *g*↔*e*′ electrostatic interactions can also form interhelical salt bridges that affect dimerization specificity and stability [62]. For investigating the contribution of charged residues at the *e* and *g* positions in governing dimerization properties of *Arachis* bZIP proteins, the frequencies of attractive and repulsive *g*↔*e*′ pairs in each heptad was calculated (Fig. 4c). Across all heptads, the attractive *g*↔*e*′ pairs were concentrated in the second (15.6%), fifth (35%) and sixth (30%) heptads, indicating they can form complete attractive *g*↔*e*′ interactions and contribute to stability through complementation in a heterodimer. Three groups comprising 28 subfamilies (BZ1–BZ28) were further divided based on homo- and heterodimerization properties, particularly dimerization specificity [60, 63] (Additional file 7).

### The impact of whole genome duplication and tandem duplication on the expansion of *Arachis bZIP* gene family

**Fig. 5:**
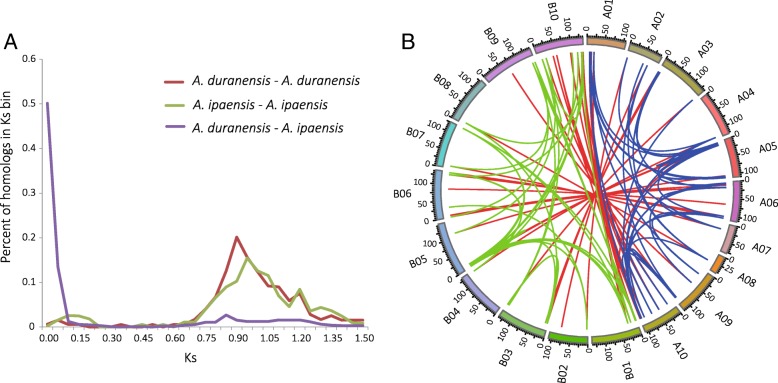
Whole genome duplication (WGD)-derived *Arachis bZIP* genes. **a** The Ks distribution of paralogs from WGD-derived duplicated genomic blocks in *A. duranensis* and *A. ipaensis*. **b** The duplicated *bZIP* paralogs derived from WGD were linked by blue (in *A. duranensis*) and green (in *A. ipaensis*) lines. The *bZIP* orthologs between *A. duranensis* and *A. ipaensis* were linked by read lines

We identified the genome-wide collinear duplicated blocks in the *A. duranensis* and *A. ipaensis* genomes and the orthologous collinear blocks between two genomes. The pairwise synonymous distances (Ks values) between the paralogs and orthologs within collinear blocks were calculated, and their frequency distributions were plotted (Fig. 5a; Ks bin = 0.05). The peak Ks frequency between *A. duranensis* and *A. ipaensis*, representing average sequence variation, was 0.035. This represented the sequence divergence between these two closely related *Arachis* species, which was estimated to have diverged ~ 2.16 million years ago [35]. Further, the Ks peaks for *A. duranensis* and *A. ipaensis* paralogs were 0.90 and 0.95, respectively, corresponding to the sequence divergence of early papilionoid whole genome duplication (WGD) event occurred ~ 58 million years ago [35].

We detected 35 *AdbZIPs* and 32 *AibZIPs* involved in duplicated genomic blocks, accounting for around 70% (35/50) and 71% (32/45) of the *bZIP* genes in each species (Fig. 5b and Additional file 8). Moreover, the duplicated *bZIP* gene pairs occurred either within a chromosome or between chromosomes, and some of these pairs were segmentally duplicated once, twice, or three times. This result indicated preferential gene retention and frequent chromosomal arrangements after WGD. Tandem duplications were detected for only two gene pairs (*AdbZIP33*/*AdbZIP34* and *AdbZIP41*/*AdbZIP42*) in *A. duranensis* and only one gene pair (*AibZIP28*/*AibZIP29*) in *A. ipaensis*. This suggested that tandem duplication occurred rarely and was not more important than segmental duplication in the expansion of the *bZIP* gene family. We also used phylogenetic and syntenic analyses to identify 35 orthologous *bZIP* gene pairs between *A. duranensis* and *A. ipaensis.* These genes were also homeologs between the two subgenomes of the tetraploid peanut.

To understand the evolutionary constraints acting on the *Arachis bZIP* genes, we calculated Ka/Ks values for each duplicated *bZIP* gene pair in two *Arachis* species (Additional file 9). For most of these pairwise comparisons, the Ka/Ks values were less than 0.5 (only one pairwise comparison between duplicated *AdbZIPs* and only two between duplicated *AibZIPs* were larger than 0.5). This suggested that strong purifying selection acted on the *Arachis* duplicated *bZIPs* to remove deleterious mutations at the protein level.

### Expression analysis of *Arachis bZIP* genes during peanut seed development

**Fig. 6:**
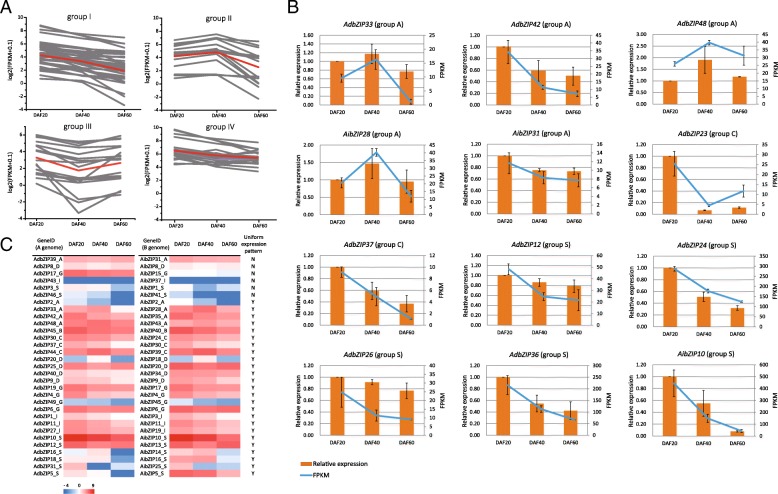
*Arachis bZIP* gene expression during peanut seed development. **a** Four groups (groups I - IV) including corresponding bZIP genes with specific expression profile were recognized. In each subgroup, the gray lines indicated the expression values of *bZIPs* at DAF20, DAF40 and DAF60. The red line show the average FPKM of all *bZIP* genes. **b** qRT-PCR verification of 12 *bZIP* genes expressed during seed development. The relative gene expression levels as measured by qRT-PCR (orange histograms) and by RNA-seq (blue lines) are shown. Results are based on three biological replicates; error bars represent SE. **c** Expression pattern of *bZIP A. duranensis* and *A. ipaensis* orthologs during seed development. The similar (denoted as Y) or diverged (denoted as N) expression pattern between orthologs were indicated

To profile *bZIP* gene expression, we used our previously published RNA-seq data [44], which documents gene expression in peanut seeds at different developmental stages: 20, 40, and 60 days after flowering (DAF). Using this data, we identified the FPKM values for all *Arachis bZIPs* and all differentially expressed *bZIPs* across the three developmental stages. With the exception of 24 *bZIPs*, which were not expressed at any developmental stage, four groups including corresponding *bZIP* genes with specific expression profile were recognized(Fig. 6a and Additional file 10). The first group comprised 37 *bZIPs* that were up-regulated during early development (20 DAF), but down-regulated thereafter (at 40 and 60 DAF). The second group comprised 15 *bZIPs* that were up-regulated at 40 DAF, while the third group comprised 17 *bZIPs* that were down-regulated at 40 DAF. The fourth group comprised 22 *bZIPs* that were highly expressed across all three developmental stages. The highly expressed *bZIPs* in group four were mainly distributed in clades A, C, and S. Several of these *bZIPs* were homologous to genes that have been implicated in seed development in other plants, such as *Arabidopsis* [4], rice [8] and maize [7]. Here, 12 *bZIPs*, which were highly expressed and homologous to previous well-studied genes in seed development, were selected for qRT-PCR confirmation, and found that the expression patterns determined by RNA-seq were consistent with those found using qRT-PCR (Fig. 6b).

In group A, *AdbZIP33* and *AibZIP28* were orthologous to *Arabidopsis* ABA insensitive 5 (*ABI5*), which is associated with ABA-signaling as well as the regulation of seed development and longevity in *Arabidopsis* [64] and legumes [27]. Our RNA-seq and qRT-PCR results showed that both orthologous *ABI5* copies from the two subgenomes of the tetraploid peanut were highly expressed during development, suggesting the function of these genes may be similar in peanut and *Arabidopsis*. Our qRT-PCR results also indicated that the group A genes *AdbZIP42*, *AdbZIP48* and *AibZIP31* were stably expressed during development (Fig. 6b and Additional file 11). These genes are homologous to *ABFs* and *AREB*, which are involved in ABA-mediated seed development, germination, and embryo maturation [65]. Three genes in group C (*AdbZIP23*, *AdbZIP37,* and *AibZIP30*) were also highly expressed, and are homologous to the maize *bZIP* factor *Opaque2*. *Opaque2* regulates protein accumulation and amino acid and sugar metabolism in maize seeds [66–69]. In addition, the group S genes *AibZIP10*, *AdbZIP12*, *AdbZIP24*, *AdbZIP26,* and *AdbZIP36* were extremely highly expressed in peanut seeds (Fig. 6b and Additional file 11). Interestingly, the group S genes *AdbZIP24* and *AdbZIP36* had a similar expression pattern to the group C genes *AdbZIP37* and *AibZIP30*: a decrease in expression level as seed development progressed.

We then further investigated the divergences in gene expression between homeologous genes from the AA and BB genomes of the tetraploid peanut. The heatmap analysis indicated that the overall expression patterns across seed development were similar for 31 pairs of homeologous/orthologous genes from the AA and BB genomes. We used the differential expression analysis method in combination with statistical methods to calculate differences in gene expression between these gene pairs for each sample. We found that 3 pairs of genes (*AdbZIP5* and *AibZIP5*, *AdbZIP17* and *AibZIP15*, *AdbZIP46* and *AibZIP41*) were differentially expressed at 20 DAF, 3 pairs (*AdbZIP3* and *AibZIP1*, *AdbZIP4* and *AibZIP4*, *AdbZIP49* and *AibZIP45*) at 40 DAF, and 5 pairs (*AdbZIP3* and *AibZIP1*, *AdbZIP33* and *AibZIP28*, *AdbZIP37* and *AibZIP30*, *AdbZIP10* and *AibZIP10*, *AdbZIP1* and *AibZIP3*) at 60 DAF. These results indicated the overall expression conservation between two genomes, but suggested that 20% of the genes had diverged in expression during the parallel evolution and polyploidization of two genomes (Fig. 6c).

### qRT-PCR expression profiles of *Arachis bZIP* genes under salt stress

**Fig. 7:**
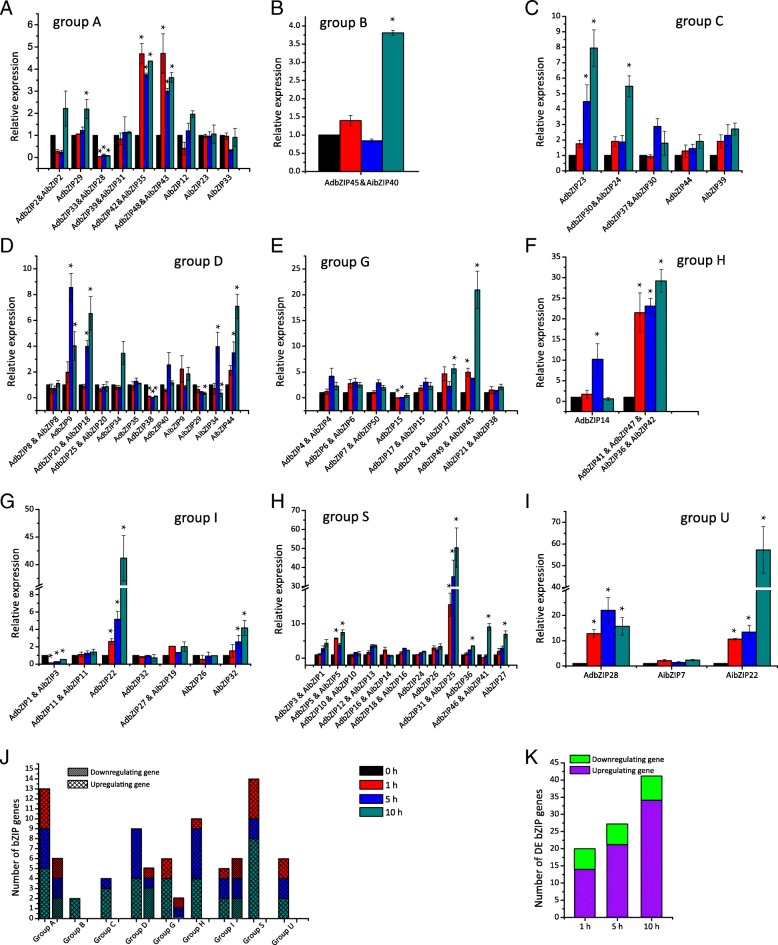
*Arachis bZIP* gene expression levels in peanut roots after 0, 1, 5, and 10 h of salt treatment. **a–i** *bZIP* gene expression levels in different groups. *: *P* < 0.05. **j** The number of significantly differently expressed *bZIP* genes in each group. **k** The number of significant differently expressed *bZIP* genes after 1, 5, and 10 h of salt treatment

We used qRT-PCR to explore changes in *bZIP* gene expression in response to salt-treatment (Fig. 7 and Additional file 12). We were unable to clearly amplify 4 *bZIPs* with PCR. After peanut roots were treated with salt for 1 h, 20 genes were significantly differentially expressed; after 5 h, 27 genes were significantly differentially expressed; and after 10 h, 41 genes were significantly differentially expressed (Fig. 7j; Student’s *t* test: *P* < 0.05). At each time point, many more genes were up-regulated than were down-regulated (14 vs. 6 at 1 h; 21 vs. 6 at 5 h; and 34 vs. 7 at 10 h). Among these differentially expressed *bZIPs* after salt treatment, many of them were distributed in groups A and S (Fig. 7k), indicating *bZIPs* in these groups play important roles in sugar signaling and abiotic stress regulation [4, 70, 71].

Group A *bZIPs* possess the CKII and Ca2 + −dependent protein kinase phosphorylation site motifs involved in stress and/or ABA signaling, and these motifs are important for plant adaptation to various abiotic environmental stressors [72]. Indeed, many group A genes are associated with the salt stress response. In *Arabidopsis*, *ABI5* and *ABFs*/*AREB* are key ABA-dependent signal transduction factors involved in abiotic stress tolerance [22, 73]. The over-expression of *GhABF2* significantly improved salt stress tolerance both in *Arabidopsis* and cotton [74]. In tomato, *slAREB1* and *slbZIP1* knockout increased salt stress tolerance, while *slAREB1* and *slbZIP1* over-expression reduced salt stress tolerance [75, 76]. Here, genes *AdbZIP42* and *AibZIP35* were significantly up-regulated in response to salt stress, and these genes are homologous to *ABFs*, *GhABF2*, *slAREB1*, and *slbZIP1*. In addition, these genes have been reported to be phosphorylated by the ABA-activated SnRK2 protein kinases [77–80], suggesting phosphorylating ABA response element-binding factors may be critical for the ABA-mediated salt stress response.

The group B genes *AdbZIP45* and *AibZIP40* were up-regulated after 10 h of salt stress, and these genes are homologous to *AtbZIP17,* which could improve the expression of several salt stress response genes in *Arabidopsis* [28]. Seven group G *bZIP* genes (*AdbZIP7*, *AdbZIP15*, *AdbZIP19*, *AdbZIP50*, *AibZIP17*, *AibZIP21*, and *AibZIP38*) were homologous to *Arabidopsis AtbZIP41* and tomato *slbZIP38*, and these genes have both been shown to negatively regulate salt stress [29]. Of these seven genes, *AdbZIP15* was significantly down-regulated after 1 h and 5 h of salt stress treatment, while *AdbZIP19* and *AibZIP17* were significantly up-regulated after 10 h of salt stress. Thus, *AdbZIP15*, *AdbZIP19* and *AibZIP17* might confer resistance to salt stress. *AdbZIP15* might be a negative regulator of salt stress, as its expression pattern was similar to that of *slbZIP38* in response to salt stress.

The group S genes *AdbZIP24* and *AdbZIP36* were homologous to *AtbZIP1*, *AtbZIP53*, *MtbZIP2*, and *MtbZIP26,* and the expression patterns of these genes in response to salt stress were similar (Fig. 7). In particular, *AdbZIP36* was significantly up-regulated after 10 h of salt stress. Two homologous genes in *Arabidopsis*, *AtbZIP1* and *AtbZIP53*, were shown to reprogram the primary carbohydrate and amino acid metabolism to help roots adapt to salt stress [30]. The homologs *MtbZIP2* and *MtbZIP26* are also transcriptionally induced by salt treatment, and improve plant tolerance to salt stress [32]. Notably, the expression pattern of *AdbZIP36* was similar to those of *AtbZIP1*, *MtbZIP2*, and *MtbZIP26* in *Arabidopsis* and *M. truncatula* [30, 32], suggesting that *AdbZIP36* might be a positive regulator of tolerance to salt stress in the peanut. In summary, our study of expression analysis has identified several candidate peanut bZIPs, which may be associated with the salt-stress response, as targets for future research.

## Conclusions

Despite the importance of bZIP transcription factors for plant growth, development, and abiotic stress responses, little is known about the *bZIP* gene family in peanut. Here, we used the previously published peanut reference genome to perform a comprehensive analysis of peanut *bZIPs*, including sequence identification, phylogenetic construction, motif composition characterization, gene structure analysis, and determination of DNA-binding-site specificity and dimerization properties. We also investigated evolutionary expansion of the *bZIP* gene family. *bZIP* genes were clearly divided into phylogenetic clades. These clades were supported by various group-specific sequence characteristics, including exon/intron structure, intron phases in domain, MEME motif composition, DNA-binding specificity, and dimerization properties. By analyzing changes in *bZIP* gene expression during seed development and in response to salt stress, we characterized the overall expression patterns for different groups of *bZIPs*. We also identified several candidate bZIP proteins that may be important for seed development and the salt stress response. The information generated in this study could facilitate further research on *bZIP* gene family and other gene families in peanut.

## Additional files

Additional file 1: Identified bZIP proteins in peanuts and related information. (XLSX 18 kb)

Additional file 2: Positions and patterns of introns within the basic and hinge regions of the bZIP domains of the *Arachis bZIP* transcription factors. (PDF 327 kb)

Additional file 3: The phylogenetic tree of bZIP genes from *Arabidopsis thaliana*, *Arachis duranensis*, *Arachis ipaensis*, and *Glycine max*. (PDF 683 kb)

Additional file 4: Map of intron-exon arrangements for the *Arachis bZIP* genes. (PDF 980 kb)

Additional file 5: MEME motif composition of the *Arachis* bZIP proteins. (PDF 520 kb)

Additional file 6: Alignment of the basic and hinge regions of 95 *Arachis* bZIP proteins. (XLSX 18 kb)

Additional file 7: Amino acid sequence alignment of the leucine zipper region of 95 *Arachis* bZIP proteins for prediction of dimerization properties. (PDF 894 kb)

Additional file 8: Chromosomal distributions of the *Arachis bZIP* genes. (PDF 512 kb)

Additional file 9: The Ka, Ks and Ka/Ks values for duplicated bZIP gene pairs in two *Arachis* genomes. (XLSX 14 kb)

Additional file 10: Four groups including corresponding bZIP genes with specific expression profile were recognized. (XLSX 5042 kb)

Additional file 11: Phylogenetic analysis of some *Arachis* bZIP proteins and their homologs in different plant species. (PDF 182 kb)

Additional file 12: Gene-specific primers used for qRT-PCR. (PDF 463 kb)

## Abbreviations

ABA: Abscisic acid
ABF: ABRE binding factors
ABI5: A insensitive 5
AREB: ABA-responsive element binding proteins
bZIP: Basic leucine zipper
DAF: Days after flowering
DE: Differential expression
DOG: Delay of germination
ER: Endoplasmic reticulum
HMM: Hidden markov model
Leu: Leucine
MEME: Multiple Em for Motif Elicitation
ML: Maximum likelihood
ORFs: Open reading frames
qRT-PCR: Quantitative real-time PCR
TF: Transcription factor
WGD: Whole genome duplication
