# Supplementary material for: Genome-wide systematic characterization of bZIP transcription factors and their expression profiles during seed development and in response to salt stress in peanut
Source: BMC Genomics. 2019 Jan 16;20:51. doi: 10.1186/s12864-019-5434-6 (PMC6335788; doi:10.1186/s12864-019-5434-6)
Supplement: Supplementary file 2 — Positions and patterns of introns within the basic and hinge regions of the bZIP domains of the Arachis bZIP transcription factors. (PDF 327 kb) [file 12864_2019_5434_MOESM2_ESM.pdf]

Additional file 2. Position and pattern of introns within the basic and hinge regions of bZIP domains of the peanut bZIP transcription factors.

Group	bZIP No.	Basic and hinge regions (intron insertion site)	Pattern (a - d) of intron phase in Figure 2
A	AdbZIP13	QGIDRKQKRMAKNRESAAKSRAKKQ AYIEKL	a
	AdbZIP29	KTVERRQKRMIKNRESAARSRARKQ AYTQEL	
	AdbZIP33	VVVERRQRRMLKNRESAARSRARRQ AYTVEL	
	AdbZIP39	KAAQQRQRRMIKNRESAARSRERKQ AYQVEL	
	AdbZIP42	KVVERRQRRMIKNRESAARSRARRQ AYTFEL	
	AdbZIP48	KTVERRQKRMIKNRESAARSRARKQ AYTTEL	
	AdbZIP2	RICENISKRMMKNRESAARSRARKQ AYTHEL	
	AibZIP12	KATLQKQRRMIKNRESAARSRERKQ AYTTDL	
	AibZIP2	RICENISKRMMKNRESAARSRARKQ AYTHEL	
	AibZIP23	KTVERRQKRMIKNRESAARSRARKQ AYTQEL	
	AibZIP28	VVVERRQRRMLKNRESAARSRARRQ AYTVEL	
	AibZIP31	KAAQQRQRRMIKNRESAARSRERKQ AYQVEL	
	AibZIP35	KVVERRQRRMIKNRESAARSRARRQ AYTFEL	
	AibZIP43	KTVERRQKRMIKNRESAARSRARKQ AYTTEL	
	AibZIP33	KASLQKQRRMIKNRESAARSRERKQAYTLEL	d
B	AdbZIP45	DDDEKRKARLMRNRESAQLSRQRKKHYVEEL	d
	AibZIP40	DDDEKRKARLMRNRESAQLSRQRKKHYVEEL	
C	AdbZIP23	PTDAKR VRRMLSNRESARRSRRRKQAHLTEL	c
	AdbZIP30	PIDMKR LRRKVSNRESARRSRRRKQAYLTDL	
	AdbZIP37	PADVKR VRRMLSNRESARRSRRRKQAHLNEL	
	AdbZIP44	PVDVKR LRRKESNRESARRSRRRKQAHLADL	
	AibZIP24	MKR LRRKVSNRESARRSRRRKQAYLTDL	
	AibZIP30	PADVKR VRRMLSNRESARRSRRRKQAHLNEL	
	AibZIP39	PVDVKR LRRKESNRESARRSRRRKQAHLADL	
D	AdbZIP20	AVDQK TMRRLAQNREAARKSRLRKK AYVQQL	b
	AdbZIP25	KADQK TLRRLAQNREAARKSRLRKK AYVQQL	
	AdbZIP34	PLDAK TLRRLAQNREAARKSRLRKK AYVQQL	
	AdbZIP35	KTDQK SLRRLAQNREAARKSRLRKK AYVQQL	
	AdbZIP38	HEDQK TLRRLAQNREAARKSRLRKK AYVQQL	
	AdbZIP40	KPIDK IQRRLAQNREAARKSRLRKK AYVQQL	
	AdbZIP8	TPDPK TLRRLAQNREAARKSRLRKK AYVQQL	
	AdbZIP9	AVDAK TLRRLAQNREAARKSRLRKK AYVQQL	
	AibZIP18	AVDQK TMRRLAQNREAARKSRLRKK AYVQQL	
	AibZIP20	KADQK TLRRLAQNREAARKSRLRKK AYVQQL	
	AibZIP29	PLDAK TLRRLAQNREAARKSRLRKK AYVQQL	
	AibZIP34	KPVDK IQRRLAQNREAARKSRLRKK AYVQQL	
	AibZIP44	KTDQK TLRRLAQNREAARKSRLRKK AYVQQL	
	AibZIP8	TPDPK TLRRLAQNREAARKSRLRKK AYVQQL	
	AibZIP9	AVDAK TLRRLAQNREAARKSRLRKK AYVQQL	
G	AdbZIP15	NVTRRDRKENSLNRELARRSRLRKQ AECEEL	a
	AdbZIP17	ERELKRQRRKQSNRESARRSRLRKQ AECDEL	
	AdbZIP19	ERELKRQKRKQSNRESARRSRLRKQ AECEEL	
	AdbZIP4	ERELKRERRKQSNRDSARRSRLRKQ AETEEL	
	AdbZIP49	NDEIRKERKRLSNRESAKRSRLRKQ KECEEL	
	AdbZIP50	ERDLKRQKRKQSNRESARKSRLRKQ AECEEL	
	AdbZIP6	ERELKRERRKQSNRESARRSRLRKQ AEAEEL	
	AdbZIP7	ERDLKRQKRKQSNRESARRSRLRKQ AECEEL	
	AibZIP15	ERELKRQRRKQSNRESARRSRLRKQ AECDEL	
	AibZIP17	ERELKRQKRKQSNRESARRSRLRKQ AECEEL	
	AibZIP21	ERDLKRQKRKQSNRESARRSRLRKQ AECEDL	
	AibZIP38	ERDLKRQKRKQSNRESARRSRLRKQ AECEDL	
	AibZIP4	ERELKRERRKQSNRDSARRSRLRKQ AETEEL	
	AibZIP45	NDEIRKERKRLSNRESAKRSRLRKQ KECEEL	
	AibZIP6	ERELKRERRKQSNRESARRSRLRKQ AEAEEL	
H	AdbZIP14	DKEYRR LKRLLRNRVSAQQARERKKVYVNDL	c
	AdbZIP41	DKENKR LKRLLRNRVSAQQARERKKAYLIDL	
	AdbZIP47	DKENKR LKRLLRNRVSAQQARERKKAYLIDL	
	AibZIP36	DKENKR LKRLLRNRVSAQQARERKKAYLIDL	
	AibZIP42	DKENKR LKRLLRNRVSAQQARERKKAYLIDL	
I	AdbZIP1	TIDPKR AKRILANRQSAARSKERKARYIQEL	c
	AdbZIP11	MMDPKR AKRILANRQSAARSKERKMRYISEL	
	AdbZIP22	TIDPKR AKRILANRQSDTRSKERKARYIQEL	
	AdbZIP27	MADPKR AKRILANRVSAARSKERKTRYISEL	
	AdbZIP32	TIDPKR AKRILANRQSAARSKERKARYIQEL	
	AdbZIP43	TVDPKR AKRILANRKSAARSKERRACYVVEL	
	AibZIP11	MMDPKR AKRILANRQSAARSKERKMRYISEL	
	AibZIP19	MADPKR AKRILANRVSAARSKERKTRYISEL	
	AibZIP26	TIDLKR AKRILANRQSAAHSKERKACYIQEL	
	AibZIP3	TIDPKR AKRILANRQSAARSKERKARYIQEL	
	AibZIP32	TIDPKR AKRILANRQSAARSKERKARYIQEL	
	AibZIP37	TVDPKR AKRILANRKSAARSKERRACYVVEL	
S	AdbZIP10	LMDQRKRKRMISNRESARRSRMRKQKHLDDL	d
	AdbZIP12	IMDQRKRKRMQSNRESARRSRMRKQQHLDSL	
	AdbZIP16	ILNERKHRRMISNRESARRSRMRKQKHLDEL	
	AdbZIP18	VMNERKRRRKISNRESARRSRMRKQRHLENL	
	AdbZIP21	VIDERKQRRMISNRESARRSRMRKQKHLDEL	
	AdbZIP24	VLDERKRKRMLSNRESARRSRMRKQKQLEDL	
	AdbZIP26	LMDQKKRKRKQSNRESARRSRMRKQKHLDDL	
	AdbZIP3	MISNRESARRSRMRKQKHLDEL	
	AdbZIP31	SSHERKIRRKQSNRESARRSRWRKKRHLENL	
	AdbZIP36	VMDERKRKRMLSNRESARRSRMRKQKQLEDL	
	AdbZIP46	LINERKHRRMISNRESARRSRMRKQRHLDEL	
	AdbZIP5	VIDERKRRRMLSNRESARRSRMRKQRHLENL	
	AibZIP1	IIDERRQRRMISNRESARRSRMRKQKHLDEL	
	AibZIP10	LMDQRKRKRMISNRESARRSRMRKQKHLDDL	
	AibZIP13	TMDQRKRKRMQSNRESARRSRMRKQQHLDSL	
	AibZIP14	ILNERKHRRMISNRESARRSRMRKQKHLDEL	
	AibZIP16	VMDERKRRRKISNRESARRSRMRKQRHLENL	
	AibZIP25	STHERKIRRKQSNRESARRSRWRKKRHLENL	
	AibZIP27	SSEERKLRRMQSNRESARRSRGRKKKHMENL	
	AibZIP41	LINERKHRRMISNRESARRSRMRKQRHLDEL	
	AibZIP5	VIDERKRRRMLSNRESARRSRMRKQRHLENL	
U	AdbZIP28	EKEARRIRRILANRESARQTIRRRQ ALCEDL	a
	AibZIP22	EKEARRIRRILANRESARQTIRRRQ ALCEDL	
	AibZIP7	EPVSKK LLRKMRNRDAAARSRERKTKYVKDL	c
