# Supplementary material for: Genome-wide systematic characterization of bZIP transcription factors and their expression profiles during seed development and in response to salt stress in peanut
Source: BMC Genomics. 2019 Jan 16;20:51. doi: 10.1186/s12864-019-5434-6 (PMC6335788; doi:10.1186/s12864-019-5434-6)

Additional file 3. The phylogenetic tree of bZIP genes from *Arabidopsis thaliana*, *Arachis duranensis*, *Arachis ipaensis*, and *Glycine max*. The phylogenetic tree was constructed using maximum likelihood method and 100 replicates were used for obtaining bootstrap values.

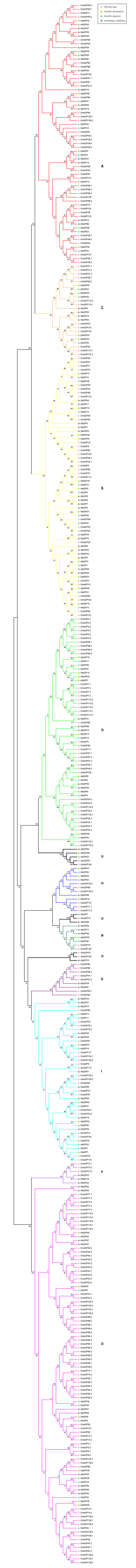

Supplement: Supplementary file 3 — The phylogenetic tree of bZIP genes from Arabidopsis thaliana, Arachis duranensis, Arachis ipaensis, and Glycine max. (PDF 683 kb) [file 12864_2019_5434_MOESM3_ESM.pdf]
