# Supplementary material for: Genome-wide systematic characterization of bZIP transcription factors and their expression profiles during seed development and in response to salt stress in peanut
Source: BMC Genomics. 2019 Jan 16;20:51. doi: 10.1186/s12864-019-5434-6 (PMC6335788; doi:10.1186/s12864-019-5434-6)

Additional file 4. The map of intron-exon arrangement of peanut bZIP genes.

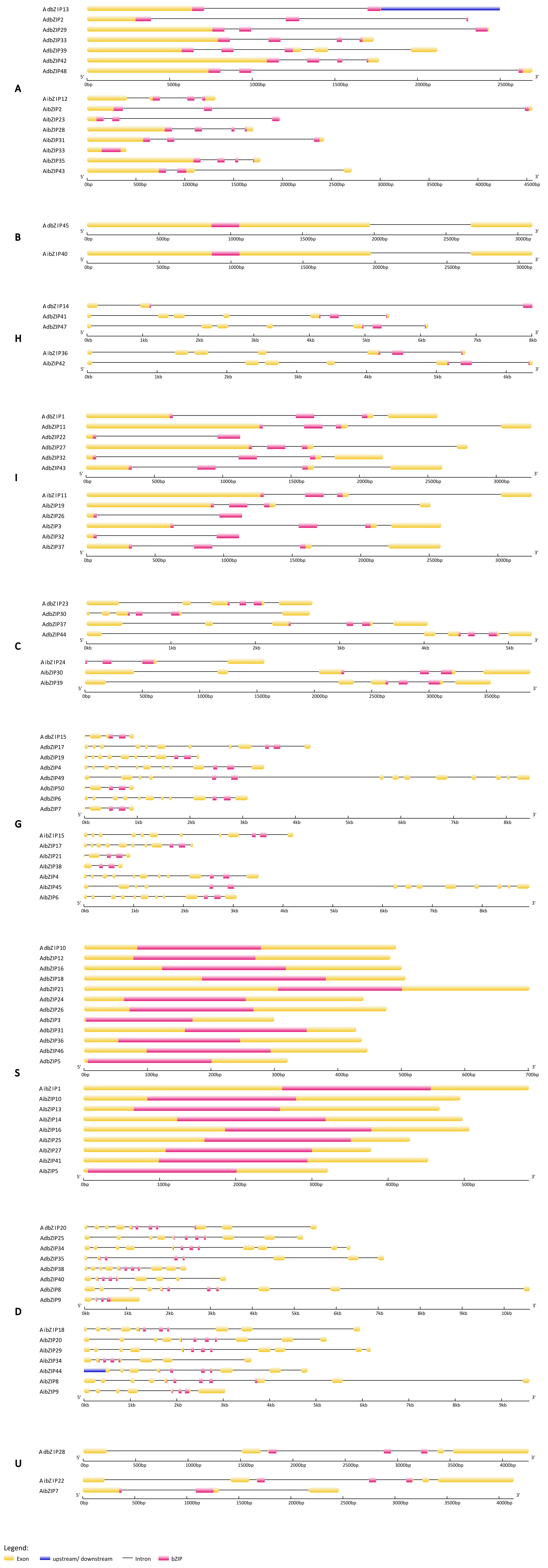

Supplement: Supplementary file 4 — Map of intron-exon arrangements for the Arachis bZIP genes. (PDF 980 kb) [file 12864_2019_5434_MOESM4_ESM.pdf]
