# Supplementary material for: Genome-wide systematic characterization of bZIP transcription factors and their expression profiles during seed development and in response to salt stress in peanut
Source: BMC Genomics. 2019 Jan 16;20:51. doi: 10.1186/s12864-019-5434-6 (PMC6335788; doi:10.1186/s12864-019-5434-6)
Supplement: Supplementary file 5 — MEME motif composition of the Arachis bZIP proteins. (PDF 520 kb) [file 12864_2019_5434_MOESM5_ESM.pdf]

Additional file 5. Alignment of basic and hinge regions of 95 peanut bZIP proteins. Conserved amino acids are boxed in red. The different amino acid residues at -10 and -18 positions like K and I are colored in blue.

BASIC REGION | HINGE

Positions: -40 -25 -20 -15 -10 -5 -1

GROUP	bZIP No.	Sequence
	AdbZIP13	QGIDRKQKRMAKNRESAAKSRAKKQAYIEKL
	AdbZIP29	KTVERRQKRMIKNRESAARSRARKQAYTQEL
	AdbZIP33	VVVERRQRRMLKNRESAARSRARRQAYTVEL
	AdbZIP39	KAAQQRQRRMIKNRESAARSRERKQAYQVEL
	AdbZIP42	KVVERRQRRMIKNRESAARSRARKQAYTFEL
	AdbZIP48	KTVERRQKRMIKNRESAARSRARKQAYTTEL
	AibZIP12	KATLQKQRRMIKNRESAARSRERKQAYTTDL
A	AibZIP2	RICENISKRMMKNRESAARSRARKQAYTHEL
	AibZIP23	KTVERRQKRMIKNRESAARSRARKQAYTQEL
	AibZIP28	VVVERRQRRMLKNRESAARSRARRQAYTVEL
	AibZIP31	KAAQQRQRRMIKNRESAARSRERKQAYQVEL
	AibZIP35	KVVERRQRRMIKNRESAARSRARKQAYTFEL
	AibZIP43	KTVERRQKRMIKNRESAARSRARKQAYTTEL
	AibZIP33	KASLQKQRRMIKNRESAARSRERKQAYTLEL
	AdbZIP2	RICENISKRMMKNRESAARSRARKQAYTHEL
B	AdbZIP45	DDDEKRKARLMRNRESAQLSRQRKKHYVEEL
	AibZIP40	DDDEKRKARLMRNRESAQLSRQRKKHYVEEL
	AdbZIP23	PTDAKRVRRMLSNRESARRSRRRKQAHLTEL
	AdbZIP30	PIDMKRLRRKVSNRESARRSRRRKQAYLTDL
	AdbZIP37	PADVKRVRRMLSNRESARRSRRRKQAHLNEL
C	AdbZIP44	PVDVKRLRRKESNRESARRSRRRKQAHLADL
	AibZIP24	---MKRLRRKVSNRESARRSRRRKQAYLTDL
	AibZIP30	PADVKRVRRMLSNRESARRSRRRKQAHLNEL
	AibZIP39	PVDVKRLRRKESNRESARRSRRRKQAHLADL
	AdbZIP20	AVDQKTMRRLAQNREAARKSRLRKKAYVQQL
	AdbZIP25	KADQKTLRRLAQNREAARKSRLRKKAYVQQL
	AdbZIP34	PLDAKTLRRLAQNREAARKSRLRKKAYVQQL
	AdbZIP35	KTDQKSLRRLAQNREAARKSRLRKKAYVQQL
	AdbZIP38	HEDQKTLRRLAQNREAARKSRLRKKAYVQQL
	AdbZIP40	KPIDKIQRRLAQNREAARKSRLRKKAYVQQL
	AdbZIP8	TPDPKTLRRLAQNREAARKSRLRKKAYVQQL
D	AdbZIP9	AVDAKTLRRLAQNREAARKSRLRKKAYVQQL
	AibZIP18	AVDQKTMRRLAQNREAARKSRLRKKAYVQQL
	AibZIP20	KADQKTLRRLAQNREAARKSRLRKKAYVQQL
	AibZIP29	PLDAKTLRRLAQNREAARKSRLRKKAYVQQL
	AibZIP34	KPVDKIQRRLAQNREAARKSRLRKKAYVQQL
	AibZIP44	KTDQKTLRRLAQNREAARKSRLRKKAYVQQL
	AibZIP8	TPDPKTLRRLAQNREAARKSRLRKKAYVQQL
	AibZIP9	AVDAKTLRRLAQNREAARKSRLRKKAYVQQL
	AdbZIP15	NVTRRDRKENSLNRELARRSRLRKQAECEEL
	AdbZIP17	ERELKRQRRKQSNRESARRSRLRKQAECDEL
	AdbZIP19	ERELKRQKRKQSNRESARRSRLRKQAECEEL
	AdbZIP4	ERELKRERRKQSNRDSARRSRLRKQAETEEL
	AdbZIP49	NDEIRKERKRLSNRESAKRSRLRKQKECEEL
	AdbZIP50	ERDLKRQKRKQSNRESARKSRLRKQAECEEL
	AdbZIP6	ERELKRERRKQSNRESARRSRLRKQAEAEEL
G	AdbZIP7	ERDLKRQKRKQSNRESARKSRLRKQAECEEL
	AibZIP15	ERELKRQRRKQSNRESARRSRLRKQAECDEL
	AibZIP17	ERELKRQKRKQSNRESARRSRLRKQAECEEL
	AibZIP21	ERDLKRQKRKQSNRESARRSRLRKQAECEDL
	AibZIP38	ERDLKRQKRKQSNRESARRSRLRKQAECEDL
	AibZIP4	ERELKRERRKQSNRDSARRSRLRKQAETEEL
	AibZIP45	NDEIRKERKRLSNRESAKRSRLRKQKECEEL
	AibZIP6	ERELKRERRKQSNRESARRSRLRKQAEAEEL
	AdbZIP14	DKEYRRLKRLLRNRVSAQQARERKKVYVNDL
	AdbZIP41	DKENKRLKRLLRNRVSAQQARERKKAYLIDL
H	AdbZIP47	DKENKRLKRLLRNRVSAQQARERKKAYLIDL
	AibZIP36	DKENKRLKRLLRNRVSAQQARERKKAYLIDL
	AibZIP42	DKENKRLKRLLRNRVSAQQARERKKAYLIDL
	AdbZIP1	TIDPKRAKRILANRQSAARSKERKARYIQEL
	AdbZIP11	MMDPKRAKRILANRQSAARSKERKMRYISEL
	AdbZIP22	TIDPKRAKRILANRQSDTRSKERKARYIQEL
	AdbZIP27	MADPKRAKRILANRVSAARSKERKTRYISEL
	AdbZIP32	TIDPKRAKRILANRQSAARSKERKARYIQEL
I	AdbZIP43	TVDPKRAKRILANRKSAARSKERRACYVVEL
	AibZIP11	MMDPKRAKRILANRQSAARSKERKMRYISEL
	AibZIP19	MADPKRAKRILANRVSAARSKERKTRYISEL
	AibZIP26	TIDLKRAKRILANRQSAAHSKERKACYIQEL
	AibZIP3	TIDPKRAKRILANRQSAARSKERKARYIQEL
	AibZIP32	TIDPKRAKRILANRQSAARSKERKARYIQEL
	AibZIP37	TVDPKRAKRILANRKSAARSKERRACYVVEL
	AdbZIP10	LMDQRKRKRMISNRESARRSRMRKQKHLDDL
	AdbZIP12	IMDQRKRKRMQSNRESARRSRMRKQQHLDSL
	AdbZIP16	ILNERKHRRMISNRESARRSRMRKQKHLDEL
	AdbZIP18	VMNERKRRRKISNRESARRSRMRKQRHLENL
	AdbZIP21	VIDERKQRRMISNRESARRSRMRKQKHLDEL
	AdbZIP24	VLDERKRKRMLSNRESARRSRMRKQKQLEDL
	AdbZIP26	LMDQKKRKRKQSNRESARRSRMRKQKHLDDL
	AdbZIP3	---------MISNRESARRSRMRKQKHLDEL
	AdbZIP31	SSHERKIRRKQSNRESARRSRWRKKRHLENL
	AdbZIP36	VMDERKRKRMLSNRESARRSRMRKQKQLEDL
S	AdbZIP46	LINERKHRRMISNRESARRSRMRKQRHLDEL
	AdbZIP5	VIDERKRRRMLSNRESARRSRMRKQRHLENL
	AibZIP1	IIDERRQRRMISNRESARRSRMRKQKHLDEL
	AibZIP10	LMDQRKRKRMISNRESARRSRMRKQKHLDDL
	AibZIP13	TMDQRKRKRMQSNRESARRSRMRKQQHLDSL
	AibZIP14	ILNERKHRRMISNRESARRSRMRKQKHLDEL
	AibZIP16	VMDERKRRRKISNRESARRSRMRKQRHLENL
	AibZIP25	STHERKIRRKQSNRESARRSRWRKKRHLENL
	AibZIP27	SSEERKLRRMQSNRESARRSRGRKKKHMENL
	AibZIP41	LINERKHRRMISNRESARRSRMRKQRHLDEL
	AibZIP5	VIDERKRRRMLSNRESARRSRMRKQRHLENL
	AdbZIP28	EKEARRIRRILANRESARQTIRRRQALCEDL
U	AibZIP22	EKEARRIRRILANRESARQTIRRRQALCEDL
	AibZIP7	EPVSKKLLRKMRNRDAAARSRERKTKYVKDL
