# Supplementary material for: Genome-wide systematic characterization of bZIP transcription factors and their expression profiles during seed development and in response to salt stress in peanut
Source: BMC Genomics. 2019 Jan 16;20:51. doi: 10.1186/s12864-019-5434-6 (PMC6335788; doi:10.1186/s12864-019-5434-6)
Supplement: Supplementary file 7 — Amino acid sequence alignment of the leucine zipper region of 95 Arachis bZIP proteins for prediction of dimerization properties. (PDF 894 kb) [file 12864_2019_5434_MOESM7_ESM.pdf]

Additional file 7. Amino acid sequence alignment of the leucine zipper region of 95 *Arachis* bZIP proteins for prediction of dimerization properties. 95 bZIP proteins are categorized into 28 subfamilies (BZ1-BZ28) with similar predicted dimerization properties. Asparagines at *a* position are colored red. The prolines and glycines are bold to indicated a potential break in the α-helix. The predicted C-terminal boundary is denotedby the symbol #, other than the natural terminals which are indicated by the symbol *. The leucine zipper region is divided into heptads (*g*, *a*, *b*, *c*, *d*, *e*, *f*) from L0 to L9 to visualize the potential *g*↔*e*' pairs. Four colors are used to differentiate between different *g*↔*e*' pairs. Attractive basic-acidic pairs (R↔E and K↔E) and colored orange, attractive acidic-basic pairs (E↔R, E↔K, D↔K, and D↔R) are blue, repulsive basic pairs (K↔K, K↔Q, Q↔K, R↔K, and R↔Q) are pink and prepulsive acidic pairs (E↔E and E↔Q) are green. If single amino acid at the positions *e* or *g* is charged, the residue is colored pink for basic amino acid and green for acidic amino acid. If the *a* or *d* positon is charged, it is colored brown.

Subfamily	bZIP No.	Group	Leucine zipper region									
			L0	L1	L2	L3	L4	L5	L6	L7	L8	L9
			gabcdef	gabcdef	gabcdef	gabcdef	gabcdef	gabcdef	gabcdef	gabcdef	gabcdef	gabcdef
BZ1	AdbZIP48	A	YTTELEN	KVSRLEE	ENEKLRK	RKELENL#	LSSAPPP	PQPRYQI	RRTSSAS	F		
	AibZIP43	A	YTTELEN	KVSRLEE	ENEKLRK	RKVRFIF	EVLFYFV#	SIPAPTS	LVPCYSQ	VMNMIVR	LSTM	
BZ2	AdbZIP29	A	YTQELEN	KVSRLEE	ENERLRR	QQEIEKV#	LPSEPPP	EPKRQLR	RTSSGPL			
	AibZIP23	A	YTQELEN	KVSRLEE	ENERLRR	QQEIEKV#	LPSEPPP	EPKRQLR	RTSSGPL			
BZ3	AdbZIP42	A	YTFELEA	EVQKLKE	INKELQK	KQEEMME	MQKNQNM#	DSSFWPL	GNKRQCL	RRTVTGP	W	
	AibZIP35	A	YTFELEA	EVQKLKE	INKELQK	KQEEMME	MQKNQNM#	DSSFWPL	GNKRQCL	RRTVTGP	W	
BZ4	AdbZIP33	A	YTVELEA	ELNVLKE	ENENLKQ	ILDEAEL	KRKQEIS	QRKHTTK#	PQKRTEK	LREIRRT	ISAAW	
	AibZIP28	A	YTVELEA	ELNVLKE	ENENLKQ	ILDEAEL	KRKQEIS	QRKHTTK	AQKRTEK	LREIRRT	ISAAW*	
BZ5	AibZIP33	A	YTLELEC	LVLQLEA	ENTRLLE	EQAQIKR	QRYNHLL#	HTIIPVI	HKRKPRP	MLRRLNS	LPW	
BZ6	AdbZIP39	A	YQVELES	LAVKLEE	ENDKLMK	EKAQCSH	HKWVYSV	ILIQSHR	SLHYCSI	TGYLLLA	ICCVLND	FIGLFV*
	AibZIP12	A	YTTDLES	NVQQLEI	ENARLLS	EEIEKAN#	GGGLDSD	VILAVLD	FSLKILA	HGFVGVG	INNTVTY	
	AibZIP31	A	YQVELES	LAVKLEE	ENDKLMK	EKLTQLI	FMQLMEK#	VIPVVEK	RRPPRFL	RRVRSLQ	W	
BZ7	AdbZIP2	A	YTHELQQ	KVKELIA	ENNRLRR	QNQQQIH	D*					
	AibZIP2	A	YTHELQQ	KVKELIA	ENNRLRR	QNQQQIV	EMGSKQD#	PNEKKGK	NKLRRSG	SI		
	AdbZIP13	A	YIEKLEN	DKSRLLK	SNRRLKK	LMKT*						
BZ8	AdbZIP45	B	YVEELEE	KVRSMHS	TIADLSS	KISFVMA	ENATLRQ	QLGVGGM#	CPPPPPV	PGMYTHA	PPMAHMP	YTWMPC
	AibZIP40	B	YVEELEE	KVRSMHS	TIADLSS	KISFVMA	ENATLRQ#	QLGVGGM	CPPPPPV	PGMYPHA	PPMAHMP	YTWMPC
BZ9	AdbZIP23	C	HLTELET	QVSQLRG	ENSSLLK	RLTDISH	KYNDAAV	DNRVLKA	DVETLRA	KVKMAEE	TVKRITG#	LNPMFP
	AdbZIP37	C	HLNELET	QVSQLRG	ENSTLLK	RLTDVSH	KYNESAV	DNRVLKA	DVETLRA	KVKMAEE	TVKRITG#	LNPVMH
	AibZIP30	C	HLNELET	QVSQLRG	ENSTLLK	RLTDVSH	KYNESAV	DNRVLKA	DVETLRA	KVKMAEE	TVKRITG#	LNPVMH
	AdbZIP30	C	YLTDLES	QVEQLRL	ENATLYK	QLTDASQ	HFRDADT	NNRVLKS	DVEALRA	KVKLAED	MVARSSF	TNLNNQL*
	AdbZIP44	C	HLADLEM	QVEKLKL	ENATLYK	QFNDVRQ	QFHEADT	NNRVLKS	DVEALRA	KVKLAED	MVTRGSV	TTLNNQL*
	AibZIP39	C	HLADLEM	QVEKLKL	ENATLYK	QFNDVRQ	QFHEADT	NNRVLKS	DVEALRA	KVKLAED	MVTRGSV	TTLNNQL#
	AibZIP24	C	YLTDLES	QVEQLRL	ENATLYK	QLTDASQ	HFRDADT	NNRVLKS	DVEALRA	KVKLAED	MVARSSF	TNLNNQL#
BZ10	AdbZIP17	G	ECDELAQ	RAEALKE	ENASLRS	EVSRIRS	EYEQLAS	ENAALKE#	RLGELPG	TDDLRSR	TMNDQPA	ADDPQQS
	AibZIP15	G	ECDELAQ	RAEALKE	ENASLRS	EVSRIRS	EYEQLAS	ENAALKE#	RLGELPG	NDDLRSR	TMNDQPA	ADDPQQS
BZ11	AdbZIP6	G	EAEELAR	KVENLTA	ENVSLKS	EINQLSE	SSEKLRV	ENTTLRE	KLKNAQL#	GPADEIV	LNNTDSN	RATPVST
	AibZIP6	G	EAEELAR	KVENLTA	ENVSLKS	EINQLSD	SSEKLRV	ENTTLRE	KLKNAQL#	GPADEIV	LNNTDSN	RATPVST
	AdbZIP19	G	ECEELQK	KVESLGN	ENRTLRE	ELQRVSE	ECEKLTA	ENNSIKE	ELERLCG#	PEAVASL	E	
	AdbZIP4	G	ETEELAR	KVESLSA	ENVLLKS	EINRLAE	SSGKMRV	ENATLGE	KLKIVQM	VQTEGII	LKGIESK#	SATPKST
	AibZIP4	G	ETEELAR	KVESLSA	ENVLLKS	EINRLTE	SSEKMRV	ENATLGE	KLKNAQM	VQTEGII	LKGIESK#	SATPMST
BZ12	AdbZIP41	H	YLIDLET	RVKDLEK	KNSELKE	RLSTLQN	ENQMLRQ	ILKNTTA	SRRGSNS	GTNAE*		
	AdbZIP47	H	YLIDLET	RVKDLEK	KNSELKE	RLSTLQN	ENQMLRQ	ILKNTTA	SRRGSNS	GTNAE*		
	AdbZIP14	H	YVNDLES	RAKDLED	KNSNLFE	RISTLIN	ENTMLRK	VTD*				
	AibZIP36	H	YLIDLET	RVKDLEK	KNSELKE	RLSTLQN	ENQMLRQ	ILKNTTA	SRRGSNS	GTNAE*		
	AibZIP42	H	YLIDLET	RVKDLEK	KNSELKE	RLSTLQN	ENQMLRQ	ILKNTTA	SRRGSNS	GTNAE*		
BZ13	AdbZIP27	I	YISELEH	KVQTLQA	ETTSLST	QFTKLQM	DNSELKS	LNNEYKL	KLQALEQ	QSQLKDA	LNETLDA	EVRRLRR*
	AibZIP19	I	YISELEH	KVQTLQA	ETTSLST	QFTKLQM	DNSELKS	LNNEYKL	KLQALEQ	QSQLKDA	LNETLDA	EVRRLRR*
BZ14	AdbZIP43	I	YVVELER	KIQVLQT	EATTLSA	QLNLFQR	DTTGLTT	ENTELRL	RLQAMEQ	QAKLCDA	LNEALKK	EVDRLKI*
	AdbZIP1	I	YIQELER	KVQTLQT	EATTLSA	QLTLYQR	DTTGLSS	ENTELKL	RLQAMEQ	QAQLRDA	LNDALMK	EVERLKI*
	AdbZIP32	I	YIQELER	KVQTLQT	EATTLSA	QLTLYQR	DTNGLST	ENTELKL	RLQAMEQ	QAQLRDA	LNEALKK	EVERLKV*
	AibZIP3	I	YIQELER	KVQTLQT	EATTLSA	QLTLYQR	DTTGLSS	ENTELKL	RLQAMEQ	QAQLRDA	LNDALMK	EVERLKI*
	AibZIP37	I	YVVELER	KIQVLQT	EATTLSA	QLNLFQR	DTTGLTT	ENTELRL	RLQAMEQ	QAKLCDA	LNEALKK	EVDRLKI*
BZ15	AdbZIP11	I	YISELEH	KVQTLQT	EATTLSA	QLTLLQR	DSVGLTN	QNSELKF	RLQSMEQ	QAKLRDA	LNEALTA	EVQRLKL*
	AibZIP11	I	YISELEH	KVQTLQT	EATTLSA	QLTLLQR	DSVGLTN	QNSELKF	RLQSMEQ	QAKLRDA	LNEALTA	EVQRLKL*
BZ16	AibZIP26	I	YIQELKR	KVQTLQI	EATTLSA	QLTLYQV	RAVHLKL	LA*				
	AdbZIP22	I	YIQELER	KVQTFQT	EATTLSV	QLTLHQV#	RAVHLKP	LA				
	AibZIP32	I	YIQELER	KVQTLQT	EATTLSA	QLTLYQV#	RAVHLKP	LA				
BZ17	AdbZIP49	G	ECEELRG	NMDNLKD	ENSSLTH	MLIRLSE	ECMDLSN	ENDSIQL#	AFYVLNP	ALIADNL	ASTITYN	SMVKLWF
	AibZIP45	G	ECEELRG	NMDNLKD	ENSSLTH	MLIRLSE	ECMDLSN	ENDSIQL#	AFYVLNP	ALIADNL	ASTITYN	SMVKMWF
BZ18	AdbZIP50	G	ECEELQR	KVESLGN	ESQTLRE	ELQIVLE	ECKKLTA	ENNSIKI	MSSNNTF	AVNSQQE	ELERLCG#	PEAIASL
	AdbZIP7	G	ECEELQR	KVESLGN	ESQTLRE	ELQIVLE	ECKKLTA	ENNSIKI	MSSNNTF	AVNSQQE	ELERLCG#	PEAIASL
	AdbZIP15	G	ECEELQR	KVESLGN	ESQTLRE	ELQIVLE	ECKKLTA	ENNSIKI	MSSNNTF	AVNSQQE	ELERLCG#	PEAVASL
BZ19	AibZIP38	G	ECEDLQK	KVELGNE	SQTLREE	LQRVLEE	CEKLTAE	NNSIKIM	SSNNTFA	VNSQQEK#	LERLCGP	EAVANL
	AibZIP21	G	ECEDLQK	KVELGNE	SQTLREE	LQRVSEE	CEKLTAE	NNSIKIM	SSNNTFA	VNSQQEK#	LERLCGP	EAVASL
BZ20	AibZIP22	U	LCEDLTR	KASNLVL	ENENLKK	EKEMALK	EYQSLET	TNKHLKA	QIAKTIN#	SEVDKAP	VEQESSM	AESSNPV
	AdbZIP28	U	LCEDLTR	KASNLVL	ENENLKK	EKDLALK	EYQSLET	TNKHLKA	QIAKTIN#	SEVAKAP	VEQESSM	AESSNPV
BZ21	AibZIP17	G	CEKLTAE	NNSIKEE#	LERLCGP	EAVASLE						
