# Supplementary material for: Genome-wide systematic characterization of bZIP transcription factors and their expression profiles during seed development and in response to salt stress in peanut
Source: BMC Genomics. 2019 Jan 16;20:51. doi: 10.1186/s12864-019-5434-6 (PMC6335788; doi:10.1186/s12864-019-5434-6)

Additional file 8. The chromosomal distribution of the ***Arachis*** bZIP genes was plotted and the bZIP gene pairs on duplicated chromosomal collinear segments were connected by black lines in each species. The orthologous bZIP genes between two species were linked byorange lines.

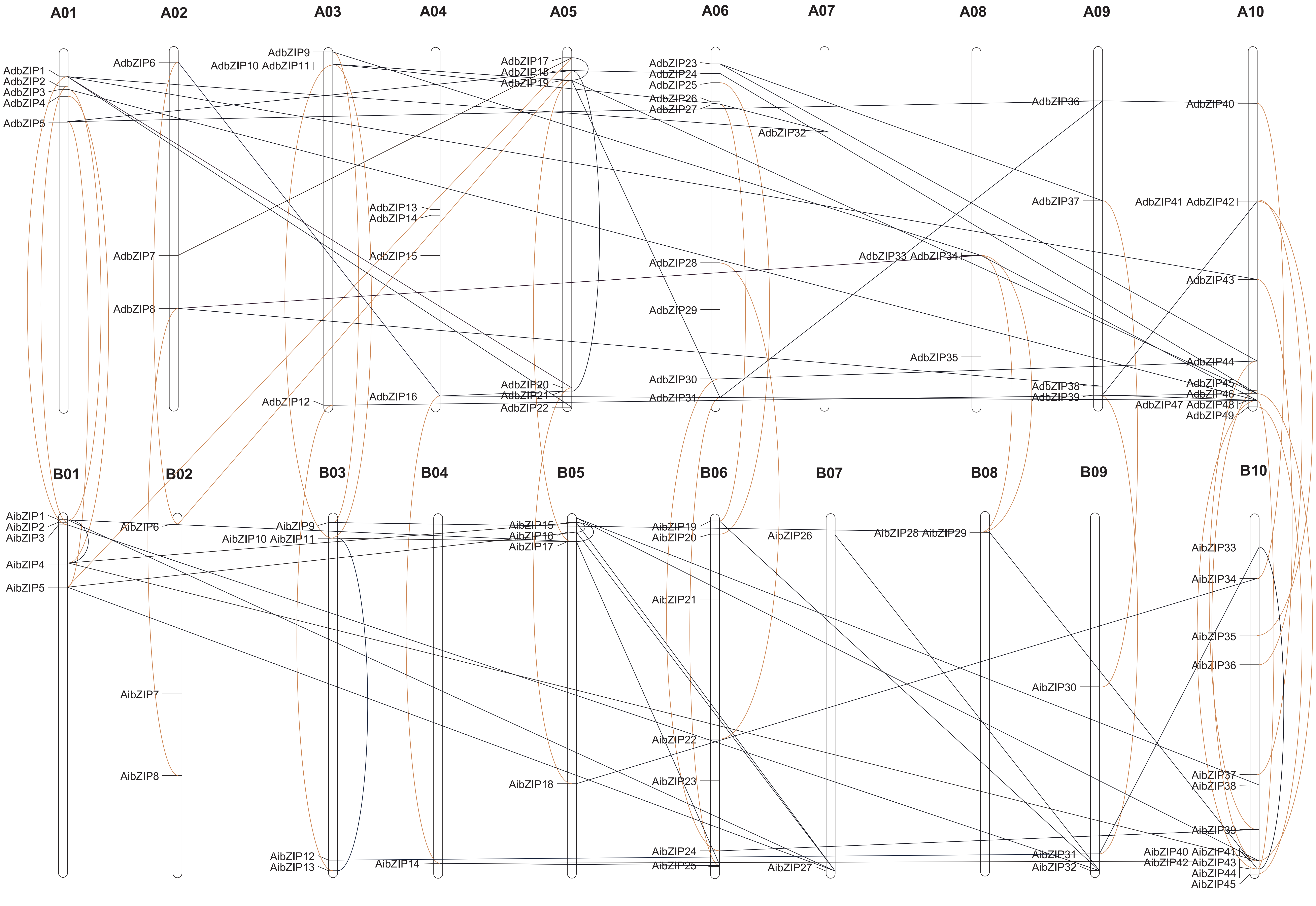

Supplement: Supplementary file 8 — Chromosomal distributions of the Arachis bZIP genes. (PDF 512 kb) [file 12864_2019_5434_MOESM8_ESM.pdf]
