# Supplementary material for: Genome-wide systematic characterization of bZIP transcription factors and their expression profiles during seed development and in response to salt stress in peanut
Source: BMC Genomics. 2019 Jan 16;20:51. doi: 10.1186/s12864-019-5434-6 (PMC6335788; doi:10.1186/s12864-019-5434-6)

Additional file 11. Phylogenetic analysis of some *Arachis* bZIP proteins and their homologs in different plant species. Analysis includes two bZIP genes, paralogs of group A, and their orthologous genes from *Arabidopsis*, tomato, cotton, and maize; seven bZIP genes, paralogs of group G, and their orthologous genes from *Arabidopsis* and tomato; and two bZIP genes, paralogs of group S, and their orthologous genes from *Arabidopsis* and cassava.

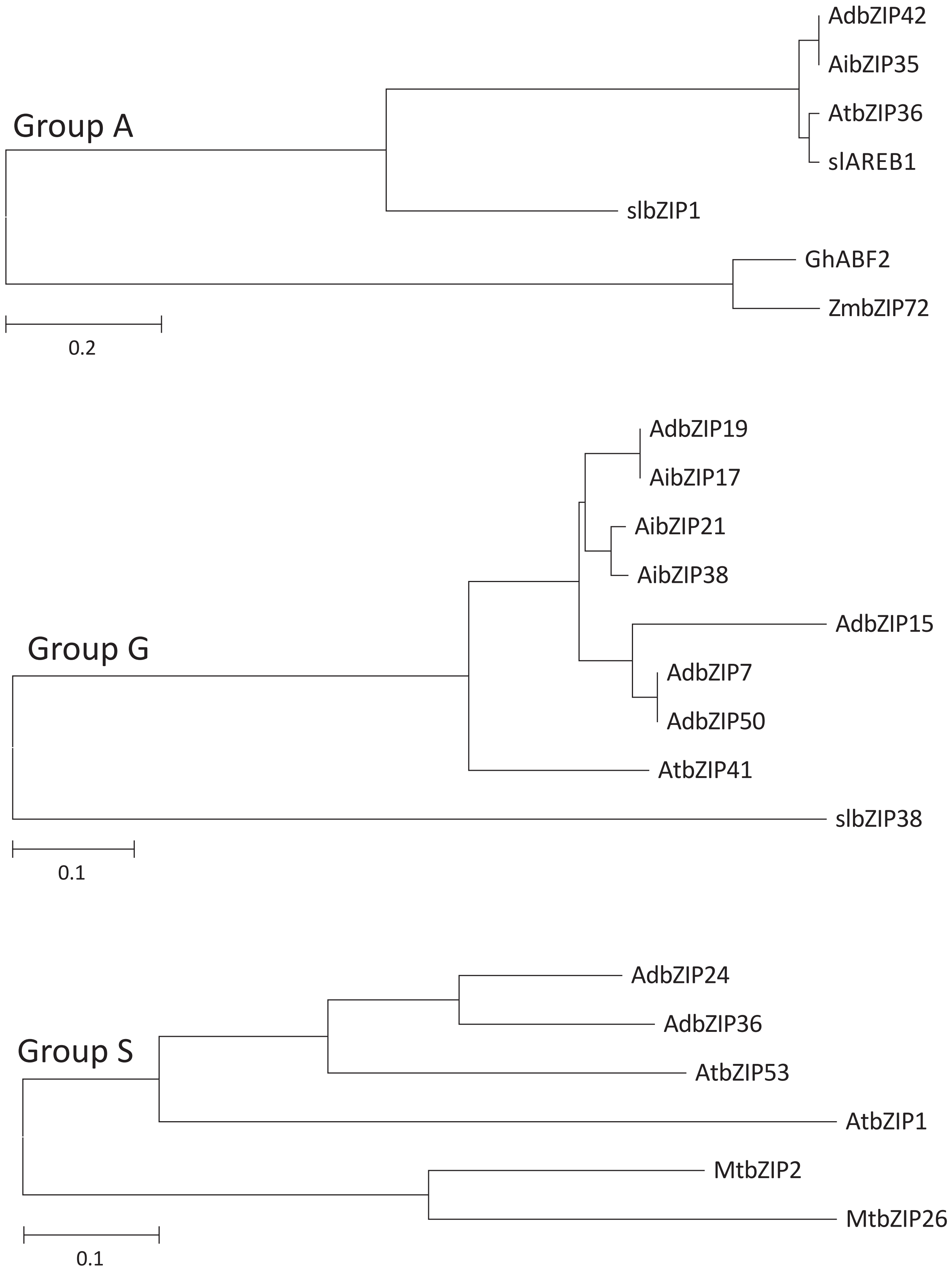

Supplement: Supplementary file 11 — Phylogenetic analysis of some Arachis bZIP proteins and their homologs in different plant species. (PDF 182 kb) [file 12864_2019_5434_MOESM11_ESM.pdf]
