# Supplementary material for: Genome-wide systematic characterization of bZIP transcription factors and their expression profiles during seed development and in response to salt stress in peanut
Source: BMC Genomics. 2019 Jan 16;20:51. doi: 10.1186/s12864-019-5434-6 (PMC6335788; doi:10.1186/s12864-019-5434-6)
Supplement: Supplementary file 12 — Gene-specific primers used for qRT-PCR. (PDF 463 kb) [file 12864_2019_5434_MOESM12_ESM.pdf]

Additional file 12. Gene-specific primers used for qRT-PCR

GeneID	Forward Primer(5'→3')	Reverse Primer(5'→3')	Product Length
AdbZIP1/AibZIP3	CTCTTTCTGCCCAATTGACC	TTCTTTCATCAATGCGTCGTT	140
AdbZIP10/AibZIP10	AGGTTCATCAACTCTTGTGCTT	CTTCCTCATCCTCGACCTCC	127
AdbZIP11/AibZIP11	CAGCTCACAATACCCCAA	TCATTCCATCTCCACCGTA	82
AdbZIP12/AibZIP13	CCACAGCAGCATATAATGGA	CTGCTGCTTCCTCATCCTC	90
AdbZIP14	CCCAACAAGCACGCGAAA	TAATCAGTTACCTTGCGGAG	145
AdbZIP15	CTCCTACTATAATGGGACGTG	TTTTCTATCTCTTCGGGTCA	134
AdbZIP16/AibZIP14	TGATGAAGCAGACGAACAAC	ACCAAAGCTCATCAAGGTGT	137
AdbZIP17/AibZIP15	AATCTTCAGCCACCAGTACCG	ACTTGATGCCATAAACCCAT	95
AdbZIP18/AibZIP16	CTAGAGAACCTGCGGAACCA	CTCAGACCGTAACCAATCGT	126
AdbZIP19/AibZIP17	TCCTAGCATGGCAACGACT	CCTTTCTTGTTTGTAGCCGAT	258
AdbZIP2/AibZIP2	CAGCAAATCTGTGCTAAC	TTAATCATGGATTTGTTGTTGA	240
AdbZIP20/AibZIP18	TCATGGCACATTATGACGAA	ATCTTGAGAAGTTCAGACGACC	148
AdbZIP22	ATCGGCAATCTGATACACGTT	TTTCAAATGGACAGCACGAA	143
AdbZIP23	GTTTTGCCCAATGCCACT	GCAGGAACTTGAGATTTTACCA	149
AdbZIP24	CCACCGATAGTGTTGGACGA	CCAGGTTCTTATTCGCCGACT	145
AdbZIP25/AibZIP20	TAAGTCAATTGCAAACTCGGAT	CTTGCATCTGCCATGTTGGAC	150
AdbZIP26	ACCGGAGTTAAGATCACA	GTGATTCAAGCCTTTTGGTA	100
AdbZIP27/AibZIP19	CCATCTTTGACCGCCAAC	CAAATTGTCCCGGTCGCTCA	150
AdbZIP28	CGGCTCATTTCACAAGCAAA	TCTCGTTTATCAATTAGGCCAA	150
AdbZIP29	CAGCACAATGTTTCTGATCAAG	AGGGCCAGCGATATAACCAG	132
AdbZIP3/AibZIP1	TTAAGGAAGAAGTCTCCGAT	TTCATCTCTACCACCGGAA	89
AdbZIP30/AibZIP24	ATATGGAAGCTGCATCACT	CATCGCTTATGACTGCATT	134
AdbZIP31/AibZIP25	GAGGCCATGTTCACACCA	TCGATCCATGTGAACCGAA	106
AdbZIP32	GAGCAACAAGCACAACTTCG	ATTTGCTCCTGTGAATGGCATC	144
AdbZIP33/AibZIP28	GACAGGCATATACGGTTGA	CTTGTTTCCTCTTGAGCTCT	105
AdbZIP34/AibZIP29	AGTAGGCCCTTCTTCATCAC	GCTTAGATTGCCTCCAACACC	283
AdbZIP35	CAGGCTTGCTCAAAATCGTG	CATCGAACTGCATTGCTCCA	200
AdbZIP36	TCCTCGAAATTGCTGAAGTCGT	GAACATGTCTGCCGAAGCCA	131
AdbZIP37/AibZIP30	TAACATCAATGGGCATACCG	TAAGATCATGACTCGCCAC	147
AdbZIP38	ACTTCAACGCGCACGTCA	ACTAATCAGCCGTTGATGCTC	142
AdbZIP39/AibZIP31	ATTCTAGAAACCCCGATCCTCC	GATCTCCCGCCACAAGTCGTC	197
AdbZIP4/AibZIP4	ACTGAAGAACTGGCACGAAA	GTCCTCACCGCTTGTATCGTT	267
AdbZIP40	AATCCCGGAAATGAAGATT	ATTCGACCAAAGCGAGCTCA	202
AdbZIP41/AdbZIP47/AibZIP36/AibZIP42	TGTCCTAGGAGTTAGTAA	CCGGTGGCTGTTAATCCA	108
AdbZIP42/AibZIP35	AGTCCCGACCAATGTAACAGC	CTTGCGAGCCCTTGACCTT	223
AdbZIP44	GCCTCATATGGCACACGTGT	CCAGCCCACCAAGACCTG	110
AdbZIP45/AibZIP40	TTCTCCTCCCCGACGCAT	TCCGATCGCCATAAACAGC	150
AdbZIP46/AibZIP41	CGTCCTACTCATACATAAGCAT	CCTTTGCTTTCTCATCCTCG	146
AdbZIP48/AibZIP43	CATAGCACAGCCATTGCACA	TCCAATATGTCCTCCGGTGT	150
AdbZIP49/AibZIP45	CAAATCAGCAGCAACAACC	TGAATCCTCGTTATTTGCAC	113
AdbZIP5/AibZIP5	TTATCGAACCGGGAATCAGC	TTAAGTTCCCGGTTTTCGAC	107
AdbZIP6/AibZIP6	GAATGCAGCCAATAAGACGA	ATGCAGGGTTCATCAACTCC	113
AdbZIP7/AdbZIP50	CCTAGGAGCTGTCAACCC	GTCTCTTCAGGTCACGTT	89
AdbZIP8/AibZIP8	ACCCAAACCTATAAAGCGAGA	CTCTATTCTGGGCAAGTCT	113
AdbZIP9	TGAACAACAGAAATGGCCTA	ATGTGGAACTCGCTCCTT	101
AibZIP12	AGACGCATGATCAAGAATCG	ACCTCCACCATTGGCTTT	150
AibZIP21/AibZIP38	CTGAGAAACAATCAACCTGC	TGTCTCTTCAGGTCACGTTC	110
AibZIP22	CTTGGATCTCTCCCGCTCT	GCTCCATCTTCATATGTCTTGC	140
AibZIP23	TTATCAGATACCCAGACTCCG	TGTAAGCCTGTTTTCTAGCAC	139
AibZIP26	CAATGCCTCCTGATAAGCTC	TTCTCTCTTTTGAATGTGCAG	102
AibZIP27	AACTGAAGAACCAGCTACTCG	GCAAAATCTGGCAAAGATCGG	123
AibZIP32	AATGCCTCCTGATAAGTTCG	TCTCTTTTGAACGTGCAG	98
AibZIP33	ACGCAAACAAGCTTACACC	AATTGAGTCTGCGAAGCATT	179
AibZIP34	GGCTACTGCAATAGAGAAG	ATTCGACCAAAGCGAGCTCA	163
AibZIP39	ACGACGACTAGTAGTCTGC	CCAGCCCACCAAGACCTG	127
AibZIP44	TCGTTTGAAGTTGACGCAAT	CTCTTCCAGCCACCTTGCAT	148
AibZIP7	TGTGCCAATTCAGTCTACGA	CTTTGTAGAGGATTGCACCC	146
AibZIP9	TTAGTAGCCACCAACAACA	CCAACCGTCTCAGTGTCT	147
